# LncRNA *MIAT* enhances cerebral ischaemia/reperfusion injury in rat model via interacting with EGLN2 and reduces its ubiquitin‐mediated degradation

**DOI:** 10.1111/jcmm.16950

**Published:** 2021-10-22

**Authors:** Suping Li, Jing Fu, Yi Wang, Chunmei Hu, Fei Xu

**Affiliations:** ^1^ Department of Neurology Sichuan Academy of Medical Sciences & Sichuan Provincial People's Hospital Chengdu China; ^2^ Department of Rehabilitation Sichuan Academy of Medical Sciences & Sichuan Provincial People's Hospital Chengdu China; ^3^ Department of Specialty of Geriatric Endocrinology Sichuan Academy of Medical Sciences & Sichuan Provincial People's Hospital Chengdu China; ^4^ Department of Otolaryngology‐Head and Neck Surgery Sichuan Academy of Medical Sciences & Sichuan Provincial People's Hospital Chengdu China

**Keywords:** EGLN2, ischaemic stroke, ischaemic/reperfusion injury, *MDM2*, *MIAT*

## Abstract

Long non‐coding RNA (lncRNA) *MIAT* (myocardial infarction associated transcript) has been characterized as a functional lncRNA modulating cerebral ischaemic/reperfusion (I/R) injury. However, the underlying mechanisms remain poorly understood. This study explored the functional partners of *MIAT* in primary rat neurons and their regulation on I/R injury. Sprague‐Dawley rats were used to construct middle cerebral artery occlusion (MCAO) models. Their cerebral cortical neurons were used for *in vitro* oxygen‐glucose deprivation/reoxygenation (OGD/R) models. Results showed that *MIAT* interacted with EGLN2 in rat cortical neurons. *MIAT* overexpression or knockdown did not alter *EGLN2* transcription. In contrast, *MIAT* overexpression increased EGLN2 stability after I/R injury via reducing its ubiquitin‐mediated degradation. EGLN2 was a substrate of MDM2, a ubiquitin E3 ligase. MDM2 interacted with the N‐terminal of EGLN2 and mediated its K48‐linked poly‐ubiquitination, thereby facilitating its proteasomal degradation. *MIAT* knockdown enhanced the interaction and reduced EGLN2 stability. *MIAT* overexpression enhanced infarct volume and induced a higher ratio of neuronal apoptosis. *EGLN2* knockdown significantly reversed the injury. *MIAT* overexpression reduced oxidative pentose phosphate pathway flux and increased oxidized/reduced glutathione ratio, the effects of which were abrogated by *EGLN2* knockdown. In conclusion, *MIAT* might act as a stabilizer of EGLN2 via reducing MDM2 mediated K48 poly‐ubiquitination. *MIAT*‐EGLN2 axis exacerbates I/R injury via altering redox homeostasis in neurons.

## 1 INTRODUCTION

Ischaemic stroke is the dominant subtype of all strokes and remains a leading cause of death and disability worldwide.^1^ Currently, thrombolysis using recombinant human tissue plasminogen activator is still the only clinically effective therapeutic strategy.^1^ However, due to the narrow therapeutic window (<4.5 h since the onset of symptoms), only a small proportion of patients received timely treatment.^1^ Besides, reperfusion is associated with complex physiological alterations (such as oxidative stress, neuroinflammation and excitotoxicity), leading to induced neuronal apoptosis and neurovascular injury.^2^ Therefore, a clear understanding of the molecular mechanisms of ischaemic/reperfusion (I/R) injury would provide new opportunities for palliative interventions.

Long non‐coding RNAs (lncRNAs) are a class of mRNA‐like transcripts than 200 nucleotides in length but lacking protein‐coding activity. They regulate many fundamental biological processes and pathophysiological events at transcriptional, post‐transcriptional and translational levels.^3^ *MIAT* (myocardial infarction associated transcript), also known as *RNCR2* (retinal non‐coding RNA 2), is a lncRNA that is involved in various diseases, such as myocardial infarction, diabetic complications, ischaemic stroke and cancers.^4^ For example, it binds to and stabilizes NF‐E2–related factor 2 (Nrf2), thereby exaggerating high glucose induced renal tubular epithelial injury.^5^ It enhances inflammation and oxidative stress in sepsis‐induced cardiac injury by sponging miR‐330‐5p and activating the downstream TRAF6/NF‐κB signalling.^6^ It promotes hypoxia/reoxygenation‐induced myocardial cell apoptosis by activating Akt/GSK‐3β signalling.^7^ Its upregulation is also observed in brain tissues after ischaemic stroke,^8^ which promotes neural cell autophagy and apoptosis via reducing ubiquitin‐mediated degradation of regulated in development and DNA damage responses 1 (NEDD1).^8^ Therefore, it might be a critical lncRNA mediating I/R injury.

Egl‐9 Family Hypoxia Inducible Factor 2 (EGLN2, also known as PHD1) is a member of the prolyl hydroxylase domain proteins (PHDs), including PHD1‐3.^9^ It is an oxygen sensor and a critical regulator of the response to hypoxia.^9^ In normoxia, PHDs mediate hydroxylation targets proteins for proteasomal degradation.^9^ One previous study showed that the PHD1 exerts a critical neuroprotective role in ischaemic stroke.^10^ PHD1^−/−^ mice had 71% reduction in infarcted size 24 h after permanent middle cerebral artery occlusion (pMCAO), without vascular changes.^10^ Cortical neurons from PHD1^−/−^ mice are less susceptible to I/R injury due to a shifting of glucose oxidation to oxidative pentose phosphate pathway (oxPPP).^10^ Therefore, EGLN2 inhibition has been considered as a potential strategy to diminish neuronal damage under the risk of cerebral ischaemia.^10^

In the current study, we investigated the functional partner of *MIAT* in primary rat neurons and identified a physical interaction between *MIAT* and EGLN2. *MIAT* increases EGLN2 stability in cortical neurons after I/R injury and modulates oxidized (GSSG) and reduced (GSH) glutathione ratio.

## 2 MATERIALS AND METHODS

### 2.1 Prediction of *MIAT* binding partners

Proteins that might interact with *MIAT* were predicted using RNAInter (http://www.rna‐society.org/rnainter/home.html).^11^ The protein sequence of rat EGLN2 was obtained from Uniprot (Q6AYU4). The possible binding sites between *MIAT* (NR_111959.1) and EGLN2 protein sequence were also predicted using the PRIdictor module of RNAInter.

### 2.2 Primary culture of rat cortical neurons

Primary cortical neurons were obtained from newborn Sprague‐Dawley rat brain tissue, according to the methods introduced previously.^12^ Briefly, the primary cortical neurons were maintained in a culture medium with 97% Neurobasal Medium, 2% B27, 1% penicillin and streptomycin (complete medium). Six days after isolations, the primary neurons were subjected to oxygen‐glucose deprivation/reoxygenation (OGD/R) treatment. The cells were washed twice and were cultured in glucose‐free DMEM. Then, the cells were maintained in a tri‐gas incubator containing 94% N_2_, 1%O_2_ and 5% CO_2_ for 2 h at 37°C. Then, the culture medium was replaced by the complete medium, and the cells were further incubated in a normal incubator for 6, 12 or 24 h.

### 2.3 Transfection and infection reagents

*MIAT* locked nucleic acid (LNA) gapmer antisense oligonucleotides (ASOs) (sequence 5′‐AAGATGTAGCATGACTC‐3′) and the scramble controls were chemically synthesized by Ribobio (Guangzhou, China).

The following adeno‐Associated Viral Vector 5 (AAV5) based AAVs were generated by Hanbio Biotechnology (Shanghai, China), including *MIAT* (NR_111959.1) overexpression (AAV5‐MIAT) (titre 3.1 × 10^12^ GC/ml), AAV5‐EGLN2 shRNA (AAV5‐shEGLN2) (titre 2.2 × 10^12^ GC/ml), AAV5‐vector control (AAV5‐vector, 5 × 10^12^ GC/ml) and AAV5‐shRNA control (titre 5.5 × 10^12^ GC/ml). The following validated sequence for the shEGLN2 were used: 5′‐GCTGCATCACCTGTATCTATT‐3′.

The following lentiviral shuttle plasmids for gene overexpression were generated based on pHBLV‐CMVIE‐IRES‐ZsGreen, including *EGLN2* (NM_001004083.1) overexpression with myc‐tag (lv‐myc‐EGLN2), *MDM2* (NM_001108099.1) overexpression with flag‐tag (lv‐flag‐MDM2), ubiquitin with HA tag (lv‐Ub‐HA) and the mutant constructs with only one lysine residue but all other mutants (K48 and K63). pLKO.1‐Puro lentiviral shuttle plasmid was used to *MDM2* shRNA (lv‐shMDM2) for gene knockdown. The following sequences for the shMDM2 were used: #1, 5′‐ GGAAATGCACCTCGTGCAATG‐3′; #2, 5′‐GCACCTCGTGCAATGAAATGA‐3′. Lentiviruses for infection were produced by co‐transfecting the shuttle plasmids and packaging plasmids (psPAX2+pMD2.G) into 293T cells as previously described.^13^ Cells were infected with lentivirus at the multiplicity of infection of 15. Proteasome inhibitor MG132 and protein synthesis inhibitor Cycloheximide (CHX, ≥99%) were purchased from Selleck (Shanghai, China).

### 2.4 Middle cerebral artery occlusion (MCAO) model in rats

Male Sprague‐Dawley rats were weighted around 220–250 g were purchased from Chengdu Dashuo Biotechnology Co., Ltd (Chengdu, China). All animal experiments were performed under the guidelines evaluated and approved by the ethics committee of Sichuan Provincial People's Hospital and in accordance with the guidelines for the Care and Use of Laboratory Animals of the National Institutes of Health. Rats were maintained in a specific pathogen free laboratory animal care facility.

Animals were randomly divided into the following groups: sham‐operated group (*N* = 10); MCAO +ASO‐NC (*N* = 25); MCAO +MIAT‐ASO (*N* = 25); MCAO +vector (*N* = 10); MCAO +MIAT (*N* = 12); MCAO +MIAT + shNC (*N* = 12); and MCAO +MIAT + shEGLN2 (*N* = 12).

GapmeRs control (ASO‐NC) and MIAT‐ASO (20 mg/kg) were intravenously (i.v.) injected via the tail vein. 48 h later, the rats were subjected to MCAO operation. For the other four groups, rats were anaesthetized and fixed to a stereotaxic apparatus (Stoelting, Kiel, WI, USA) and then were subjected to adenovirus injection. For each group, a total of 10 μl adenoviral solution was injected: vector alone group (5 × 10^9^ GC in 10 μl), MIAT group (AAV5‐MIAT: 5 × 10^9^ GC/10 μl), MIAT +shNC group (AAV5‐MIAT: 5 × 10^9^ GC/5 μl and AAV5‐shNC: 5 × 10^9^ GC/5 μl) and MIAT +shEGLN2 group (AAV5‐MIAT: 5 × 10^9^/5 μl +AAV5‐shEGLN2: 5 × 10^9^ GC/5 μl). Adenoviruses were injected into the right cerebral ventricle of rats, using a Hamilton microsyringe at bregma backwards 1 mm, 15 mm lateral and 4 mm dorsoventral. The rate of the injection was 0.5 μl/min. Subsequently, the needle was fixed for 5 min and slowly removed within 2 min. Three days post‐injection, rats were subjected to MCAO operation.

MCAO operation was established as described previously.^14^ Briefly, rats were anaesthetized by intraperitoneal (IP) injection of pentobarbital sodium (40 mg/kg). Then, a midline neck incision was performed, and the right common carotid artery (CCA), external carotid artery (ECA) and internal carotid artery (ICA) were surgically exposed. The ECA was permanently ligated. A 4–0 monofilament nylon suture with a 0.26 mm diameter rounded tip was aseptically inserted into the right CCA lumen and gently advanced into the ICA for a point 19–20 mm beyond the bifurcation of the CCA. After 90 min of occlusion, reperfusion was conducted by removing the nylon filament, followed by different time intervals of reperfusion (3, 6, 12, 18 and 24 h). The brain tissues obtained from each group were collected for the following experimental procedures.

### 2.5 2,3,5‐Triphenyltetrazolium chloride (TTC) staining

The rats were euthanized under anaesthesia 24 h after MCAO. Then, the brains were rapidly removed, cut into 2‐mm‐thick slices and stained with 2% TTC (Sigma‐Aldrich, St. Louis, MI, USA) solution at 37℃ for 30 min. The stained slices were photographed, and pale‐appearing infarcted areas were digitally analysed to calculate the infarct volume, using ImageJ software (NIH, USA).

### 2.6 qRT‐PCR analysis

RNA extraction, cDNA synthesis and qRT‐PCR were conducted according to the protocol introduced in one previous study.^15^ Gene expression was quantified by calculating fold changes using the formula 2^−ΔΔCT^ method. *GAPDH* expression served as an internal control. The primers used were provided in Table S1.

### 2.7 RNA pull‐down assay

Briefly, *MIAT* and its antisense RNA were chemically synthesized and were inserted into the sites between KpnI and SacI in pBluescript II SK+. The sense and antisense *MIAT* were transcribed *in vitro* using MAXIscript T7/T3 Transcription Kit (Thermo Fisher Scientific, Carlsbad, CA, USA). Then, the lncRNAs were labelled with biotin using a Biotin RNA Labeling Mix (Roche, Germany) and purified using an RNeasy Mini Kit (Qiagen, Valencia, CA, USA). Then, cell lysate from primary rat neurons with EGLN2 overexpression was prepared. RNA pull‐down assay was performed using a Pierce Magnetic RNA‐Protein Pull‐Down Kit (Thermo Fisher Scientific), according to the manufacturer's instruction. The retrieved protein was eluted from the RNA‐protein complex and was subjected to SDS‐PAGE. Sense *MIAT*‐specific gel bands were excised and trypsin digested. Then, the peptides were analysed by liquid chromatography tandem mass spectroscopy (LC‐MS/MS), following the method introduced previously.^16^

### 2.8 RNA immunoprecipitation (RIP)‐qPCR

RIP assays were conducted using the EZ‐Magna RIP™ RNA‐Binding Protein Immunoprecipitation Kit (Merck Millipore), according to the manufacturer's instruction. Briefly, 72 h after AAV‐EGLN2 overexpression, primary rat neurons were harvested, wash and lysed using RIPA buffer. Then, whole‐cell lysate was incubated with the RIP immunoprecipitation buffer containing protein A/G magnetic beads coated with EGLN2 antibody. Normal rat IgG was used as the control. The precipitated RNA fraction was isolated and subjected to qRT‐PCR analysis of *MIAT* expression.

### 2.9 Western blotting

Western blot was performed as described previously.^15^ In brief, total proteins were extracted from cell or tissue samples. Then, protein concentrations were determined using the BCA assay (Pierce, Rockford, IL, USA). Samples containing 30 μg protein were loaded to each lane, subjected to 12% SDS‐PAGE and transferred onto nitrocellulose membranes. The membranes were incubated consecutively with primary antibodies followed by appropriate HRP‐conjugated secondary antibodies. Protein band signals were developed using BeyoECL Star (Chemiluminescence; Beyotime, Wuhan, China) with ChampGel full automatic gel imaging system (Sage Creation Science, Beijing). The primary antibodies and dilutions were provided in Table S1.

### 2.10 Immunofluorescent staining

Primary neurons were grown on coverslips, fixed in 4% paraformaldehyde, permeabilized and blocked. Then, the cells were incubated with rabbit anti‐EGLN2 (1:50, 12984–1‐AP, Proteintech) and mouse anti‐MDM2 (1:100, 66511–1‐Ig, Proteintech) at 4°C overnight. Then, the coverslips were washed thoroughly and incubated with secondary antibodies conjugated to Alexa Fluor 647 or 488. Nuclear was stained by DAPI. Immunofluorescent staining was visualized with a fluorescence microscope (Olympus IX83, Tokyo, Japan).

### 2.11 Co‐immunoprecipitation (Co‐IP)

Co‐IP was conducted using Pierce Co‐Immunoprecipitation Kit (Thermo Scientific, Waltham, MA, USA) according to the manufacturer's instruction. In brief, primary anti‐Flag tag (10 μg) or anti‐Myc tag (10 μg) were immobilized to AminoLink Plus coupling resin. Then, primary rat neurons were collected and lysed using IP lysis buffer. The supernatant of cell lysate was collected, precleaned using control agarose resin and then was added to the spin column containing the antibody‐coupled resin. Then, the spin column was gently wobbled overnight at 4°C. Then, the spin column was centrifuged and washed. The immunoprecipitated proteins were eluted and then were subjected to Western blot analysis.

### 2.12 HE, Nissl and Terminal dexynucleotidyl transferase (TdT)‐mediated dUTP nick end labelling (TUNEL) assay

HE and Nissl staining were performed to assess morphological changes after I/R injury. Brain tissues of each group were collected, fixed with paraformaldehyde, dehydrated, dipping in wax, embedded and sectioned. Then, sections were stained with HE or cresyl violet (Nissl staining). Apoptotic neurons in the brain were visualized by TUNEL staining using an One Step TUNEL Apoptosis Assay Kit (Beyotime, Wuhan, China) following the manufacturer's instructions. Immunofluorescent images were captured under an IX83 fluorescent microscope (Olympus).

### 2.13 Oxygen consumption rate (OCR) quantitation

OCR was measured using a Seahorse XF96 (Agilent Technologies, Santa Clara, CA, USA) with the Cell Mito Stress Test Kit (Cat No. 103015–100, Agilent Technologies), following the manufacturer's instructions. OCR was normalized to the cell number as determined by CellTiter‐Glo analysis at the end of the experiments.

### 2.14 Metabolic flux assays

Oxidative pentose phosphate pathway (oxPPP) flux was measured using the isotopic non‐stationary gluconate tracer method, as described previously.^17^ Oxidized (GSSG) and reduced (GSH) glutathione ratios were measured using LC‐MS, as described previously.^18^

### 2.15 Statistical analysis

All experiments were repeated three times independently. Quantitative data were reported as means ±standard deviation (SD), based on at least three repeats of three independent tests. Unpaired *t* test with Welch's correction was used to compare the difference between two groups. One‐way ANOVA with Sidak's multiple comparisons test was conducted for multiple‐group comparison. *p* < 0.05 was considered statistically significant.

## 3 RESULTS

### 3.1 *MIAT* interacts with EGLN2 in rat cortical neurons

**FIGURE 1:**
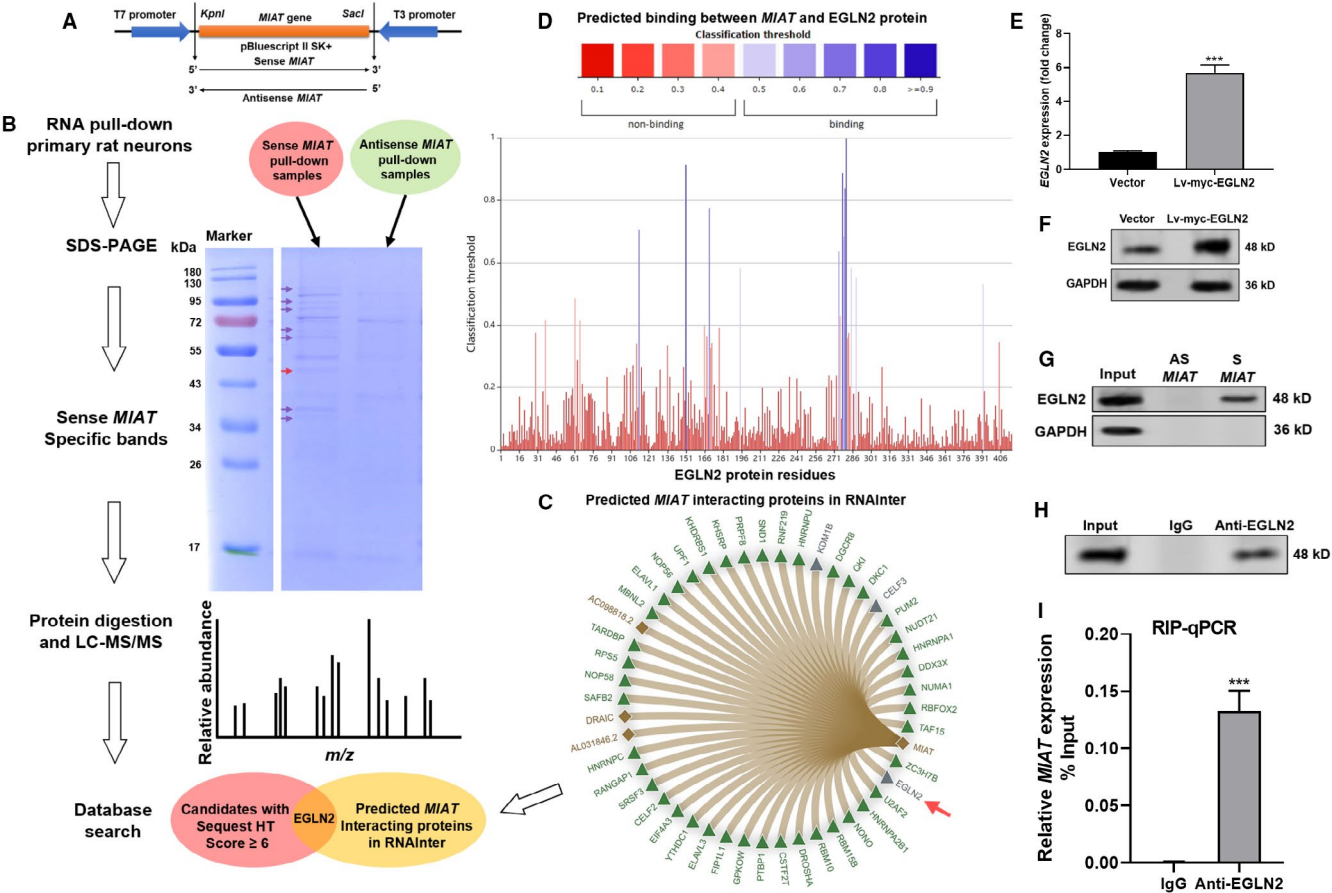
*MIAT* interacts with EGLN2 in rat cortical neurons. (A) A schematic map showing the construction of recombinant pBluescript II SK+plasmid carrying sense and antisense *MIAT*. (B) A work flow chart to identify potential *MIAT* interacting proteins, by sequential conduction of RNA pull‐down, SDS‐PAGE and LC‐MS/MS, using cellular lysate from primary rat neurons. (C) Proteins with predicted interactions with *MIAT* in RNAInter. (D) Predicted binding between *MIAT* and EGLN2 protein sequence. (E)–(F) EGLN2 mRNA (*n* = 3, E) and protein (F) expression in rat neurons with or without lv‐myc‐EGLN2 overexpression. (G) Western blot analysis to detect the presence of EGLN2 in the protein samples purified from RNA pull‐down assay using biotinylated sense (S) *MIAT* or antisense (AS) *MIAT* in rat neurons with EGLN2 overexpression. (H) Western blot analysis of the presence of EGLN2 in the anti‐EGL2 immunoprecipitated neuron lysate. (I) qRT‐PCR to detect the enrichment of *MIAT* in the anti‐EGLN2 immunoprecipitated samples. Relative enrichment was presented as the percentage of input (*n* = 3). ***, *p* < 0.001

To explore the potential functional interactor of *MIAT*, we generated biotin‐labelled sense and antisense *MIAT* by *in vitro* transcription (Figure 1A) and conducted RNA pull‐down assay in the lysates of primary rat cortical neurons (Figure 1B). Then, SDS‐PAGE was performed to separate the samples. Sense *MIAT*‐specific bands (Figure 1B, arrows) were excised, enzymatically digested, and then were subjected to LC‐MS/MS assay. The candidates identified by LC‐MS/MS were cross‐compared with the predicted *MIAT* interacting proteins by RNAInter (Figure 1C). Cross‐comparison identified ENLG2 (48 kD) as a high potential candidate (Figure 1B, 1C, red arrows). It has been characterized as an important player contributing to neuronal damage after ischaemic stroke.^10^

Bioinformatic prediction identified 12 high potential binding sites between *MIAT* and EGLN2 proteins (Figure 1D). To validate the predicted binding, RNA pull‐down assay was conducted using biotin‐labelled sense and antisense *MIAT* and the lysate of primary rat neurons with EGLN2 overexpression (Figure 1E, 1F). Western blot assay confirmed the presence of EGLN2 in the samples pulled down by sense *MIAT*, but not in the samples pulled down by antisense *MIAT* (Figure 1G). Then, RIP‐qPCR was conducted to check the enrichment of *MIAT* in the samples precipitated by anti‐EGLN2 or IgG control (Figure 1H, 1I). Results indicated that *MIAT* was significantly enriched in the anti‐EGLN2 group (Figure 1I).

### 3.2 *MIAT* increases EGLN2 stability in cortical neurons after I/R injury

**FIGURE 2:**
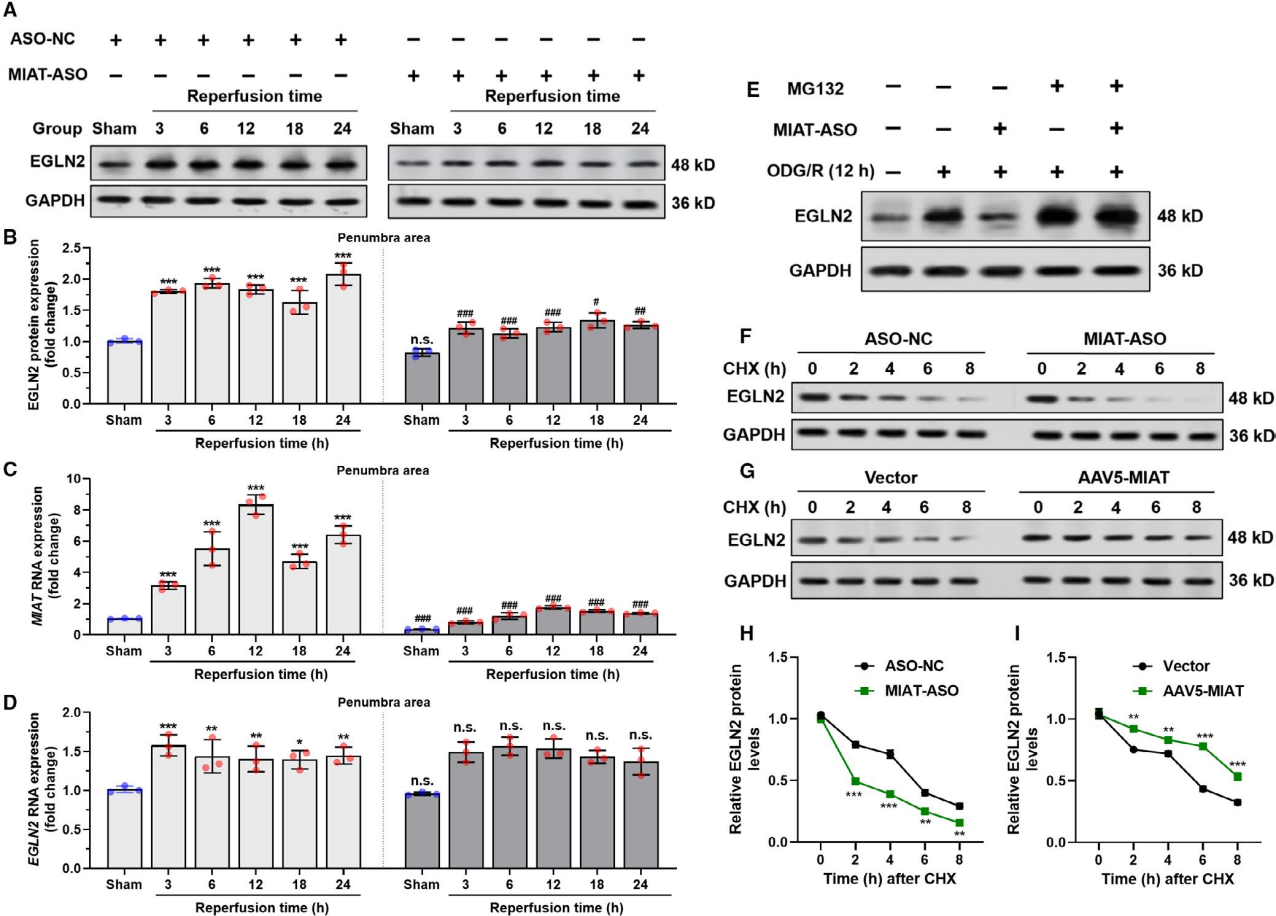
*MIAT* increases EGLN2 stability in cortical neurons after I/R injury. (A), (B). Representative images (A) and quantitative analysis (*n* = 3 per time points, (B) Immunoblot analysis showing EGLN2 protein expression in the ipsilateral brain regions of rats after I/R treatment, at the indicated time points of reperfusion. Rats were divided into two groups injected with LNA Gapmer *MIAT* ASO (MIAT‐ASO) or scrambled ASO sequence (ASO‐NC). Left panel: *, comparison was performed between the sham group (control column) and the mean of each time point after reperfusion within MIAT‐ASO groups. Right panel: **^#^** and n.s. (not significant), comparison was performed between paired columns at the same time points of reperfusion, between ASO‐NC and MIAT‐ASO groups. (C)–(D) QRT‐PCR of *MIAT* (*n* = 3, C) and *EGLN2* (*n* = 3, D) RNA expression in the corresponding tissues and time points as in panel A. Left panel: *, comparison was performed between the sham group (control column) and the mean of each time point after reperfusion within MIAT‐ASO groups. Right panel: **^#^** and n.s., comparison was performed between paired columns at the same time points of reperfusion, between ASO‐NC and MIAT‐ASO group. (E) Immunoblot analysis showing EGLN2 protein expression in primary rat neurons (with or without *MIAT* knockdown, 50 nM) 12 h after OGD/R treatment. In the MG132 group, MG132 (10 μM) was added at the beginning of reoxygenation. (F)–(I) Representative images (F)–(G) and quantitation (H‐I) of cycloheximide (CHX) pulse‐chase assays. Primary rat neurons with *MIAT* knockdown (20 nM) (F and H) or overexpression (G) and (I) cells were treated with 10 μM CHX for the indicated time, followed by Western blot analysis (*n* = 3). * and **^#^**, *p* < 0.05, ** and **^##^**, *p* < 0.01, *** and **^###^**, *p* < 0.001

In rat model of MCAO, EGLN2 protein expression was significantly elevated as early as 3 h after reperfusion and was significantly higher at all time points (3, 6, 12, 18 and 24) when compared with the sham‐operated control group (Figure 2A, 2B, left panel). At the same time, *MIAT* expression was also significantly upregulated after I/R injury (Figure 2C, left panel). *MIAT* knockdown significantly attenuated I/R induced EGLN2 upregulation at the protein level (Figure 2A, 2B, right panel), but not the mRNA level (Figure 2D).

OGD/R treatment *in vitro* induced EGLN2 upregulation, the trend of which was impaired by *MIAT* knockdown (Figure 2E). However, MG132 treatment resulted in EGLN2 accumulation and also cancelled the effect of *MIAT* knockdown (Figure 2E), suggesting that *MIAT* might influence the proteasomal degradation of EGLN2. Cycloheximide chase analysis showed that *MIAT* knockdown facilitated EGLN2 degradation (Figure 2F and 2H), while *MIAT* overexpression slowed the degradation process (Figure 2G and 2I).

### 3.3 MDM2 interacts with EGLN2 and promotes its degradation

**FIGURE 3:**
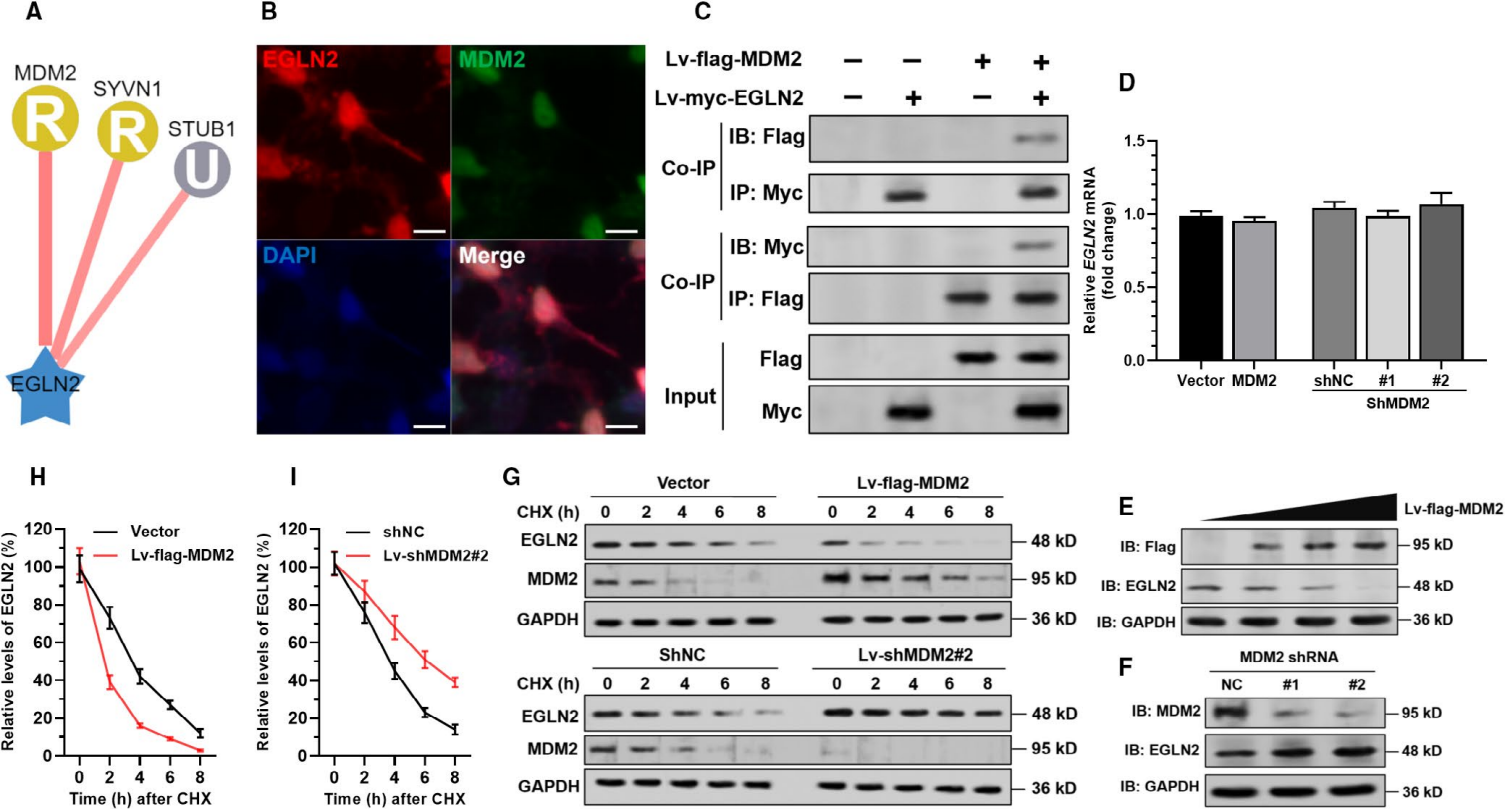
MDM2 interacts with EGLN2 and promotes its degradation. (A) Predicted E3 ligases interact with EGLN2. Confidence score, MDM2: 0.765, SYVN1: 0.736, STUB1: 0.716. The capital letter codes indicate E3 hierarchical tree, R: Ring; U: UBOX. (B) Immunofluorescent staining of EGLN2 (red), MDM2 (green) and nuclear (blue) in primary rat neurons. Scale bar=15 μm. (C) Primary neurons were coinfected with lv‐flag‐MDM2 alone or in combination with lv‐myc‐EGLN2 expression particles. IP was conducted with anti‐Myc or anti‐Flag antibodies. The potential MDM2‐EGLN2 complex was then detected by Western blot analysis with an anti‐Flag or anti‐Myc antibody. (D) QRT‐PCR analysis (*n* = 3) of *EGLN2* mRNA expression in neurons 48 h after lentiviral mediated *MDM2* overexpression or inhibition. (E), (F) Western blot analysis of EGLN2 expression in neurons with enforced MDM2 overexpression (E) or knockdown (F). (G)–(I) CHX pulse‐chase assay was performed in primary rat neurons with enforced MDM2 overexpression or knockdown. 48 h after lentiviral infection, cells were treated with 10 μM CHX for the indicated time, followed by Western blot analysis. Representative images were presented (G), and the relative EGLN2 protein levels were illustrated graphically (H)–(I) (*n* = 3)

To explore the underlying mechanisms of EGLN2 degradation, we predicted E3 ubiquitin ligases interacting with EGLN2 using UbiBrowser^19^ (http://ubibrowser.ncpsb.org/). Only the candidates with high confidence interaction score (>0.7) were identified, including murine double minute 2 (MDM2), synovial apoptosis inhibitor 1 (SYVN1) and STIP1 Homology And U‐Box Containing Protein 1 (STUB1) (Figure 3A). MDM2 is an E3 ligase involved in cerebral I/R injury (20), with the highest confidence score. IF staining confirmed the co‐localization of EGLN2 and MDM2 in primary rat neurons (Figure 3B). Then, co‐IP assay was conducted in primary neurons with enforced overexpression of flag‐MDM2 or myc‐EGLN2 (Figure 3C). Results showed that flag‐MDM2 could be immunoprecipitated by anti‐myc, while myc‐EGLN2 could be immunoprecipitated by anti‐flag (Figure 3C). MDM2 overexpression or knockdown did not alter EGLN2 transcription (Figure 3D) but negatively modulated EGLN2 expression at the protein level (Figure 3E, 3F). MDM2 overexpression decreased EGLN2 stability, while its downregulation increased EGLN2 stability (Figure 3G, 3I).

### 3.4 *MIAT* reduces MDM2 mediated K48‐linked poly‐ubiquitination of EGLN2

**FIGURE 4:**
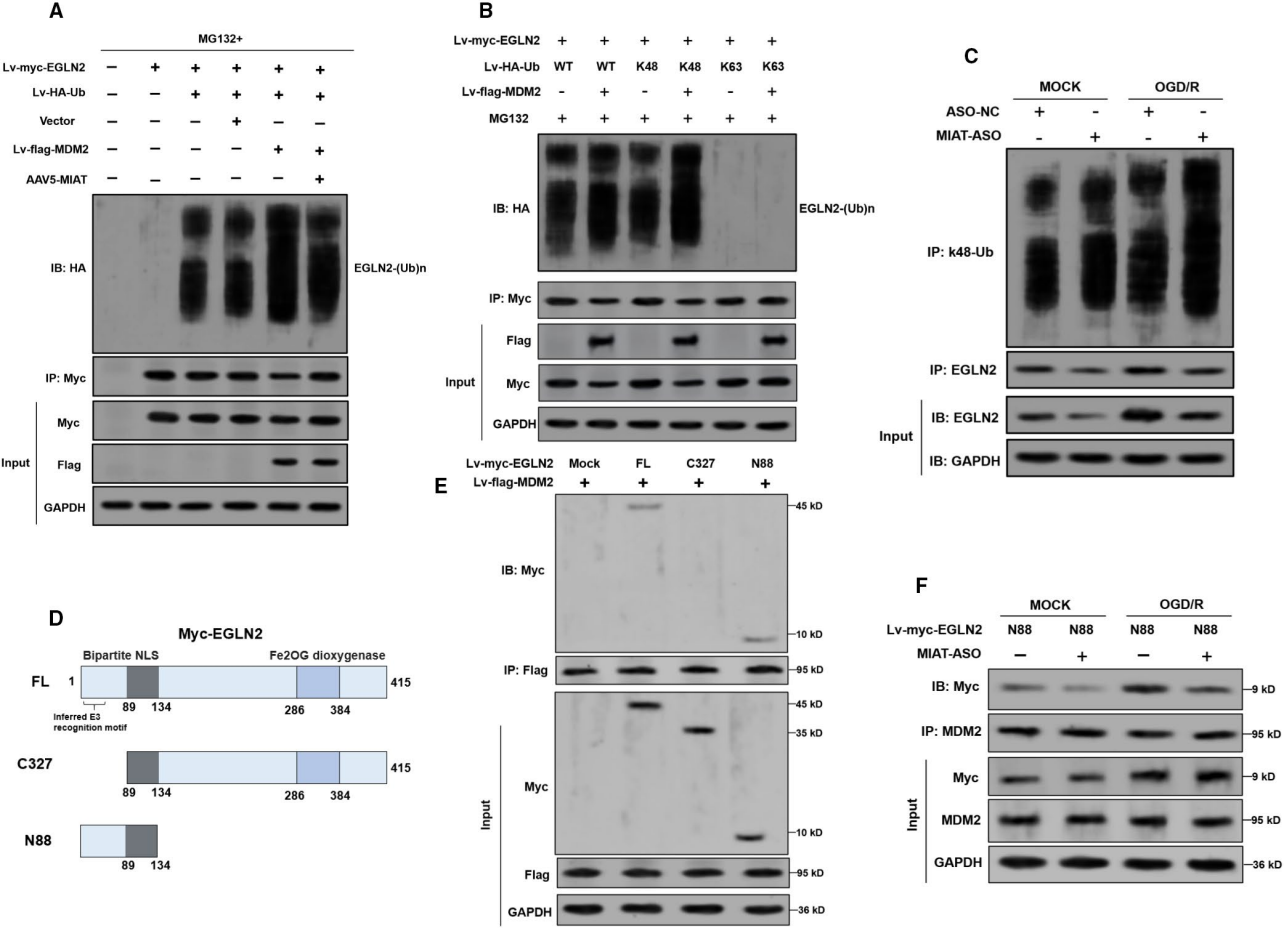
*MIAT* reduces MDM2 mediated K48‐linked poly‐ubiquitination of EGLN2. (A), (B) Primary rat neurons were subjected to infection of indicated lentiviruses/adenoviruses (Lv‐myc‐EGLN2, lv‐HA‐Ub, lv‐Flag‐MDM2 and AAV5‐*MIAT*) for 48 h, followed by treatment with MG132 (10 μM, 6 h). Then, cell lysates were immunoprecipitated with an anti‐Myc tag antibody. Ubiquitinated EGLN2 was detected by Western blot assay with an anti‐HA antibody. For treatment in panel B, mutant HA‐Ub (K48 and K63) was used as indicated. (C) Western blot analysis of K48‐linked poly‐ubiquitination of EGLN2 in *MIAT* silenced primary rat neurons after oxygen‐glucose deprivation (OGD) treatment for 12 h. (D) Schematic image showing the structure of the wild‐type full length (FL) and truncated constructs of Myc‐tag labelled EGLN2. (E) The indicated Myc‐tagged FL or truncated mutation constructs were co‐transfected with Flag‐MDM2 primary rat neurons. The MDM2/Myc‐tag complexes were immunoprecipitated by anti‐Flag antibodies. EGLN2 proteins were detected using anti‐Myc antibodies. (F) Immunoprecipitation assay was used to detect the interaction between EGLN2 N88 and MDM2 in *MIAT*‐knockdown primary rat neurons with 12 h reoxygenation after OGD treatment

MDM2 overexpression significantly increased poly‐ubiquitination of EGLN2, the effect of which was weakened by *MIAT* overexpression (Figure 4A). To identify the specific type of ubiquitin linkage catalysed by MDM2 on EGLN2, two Ub mutants with only K48 or K63 residue, but all other lysine residues replaced with arginine were generated. Primary rat neurons were coinfected with myc‐EGLN2, flag‐MDM2 and wild‐type or mutant HA‐Ub constructs. Co‐IP assay showed that MDM2 enhanced the K48 poly‐ubiquitination of EGLN2 (Figure 4B). *MIAT* knockdown also increased EGLN2 K48 poly‐ubiquitination after OGD/R treatment (Figure 4C). Bioinformatic prediction in UbiBrowser indicated that MDM2 might interact with the N terminal of EGLN2 (Figure S1). Therefore, truncated EGLN2 constructs with an N‐terminal Myc tag (Figure 4D). When the EGLN2 constructs were co‐expressed with flag‐tagged MDM2 in primary rat neurons, only the constructs containing the N terminal (FL and N88) could interact with MDM2 (Figure 4E). OGD/R treatment increased the binding of N88 with MDM2 (Figure 4F). *MIAT* knockdown weakened the binding (Figure 4F).

### 3.5 *MIAT*‐EGLN2 axis modulates I/R induced ischaemic brain damage

**FIGURE 5:**
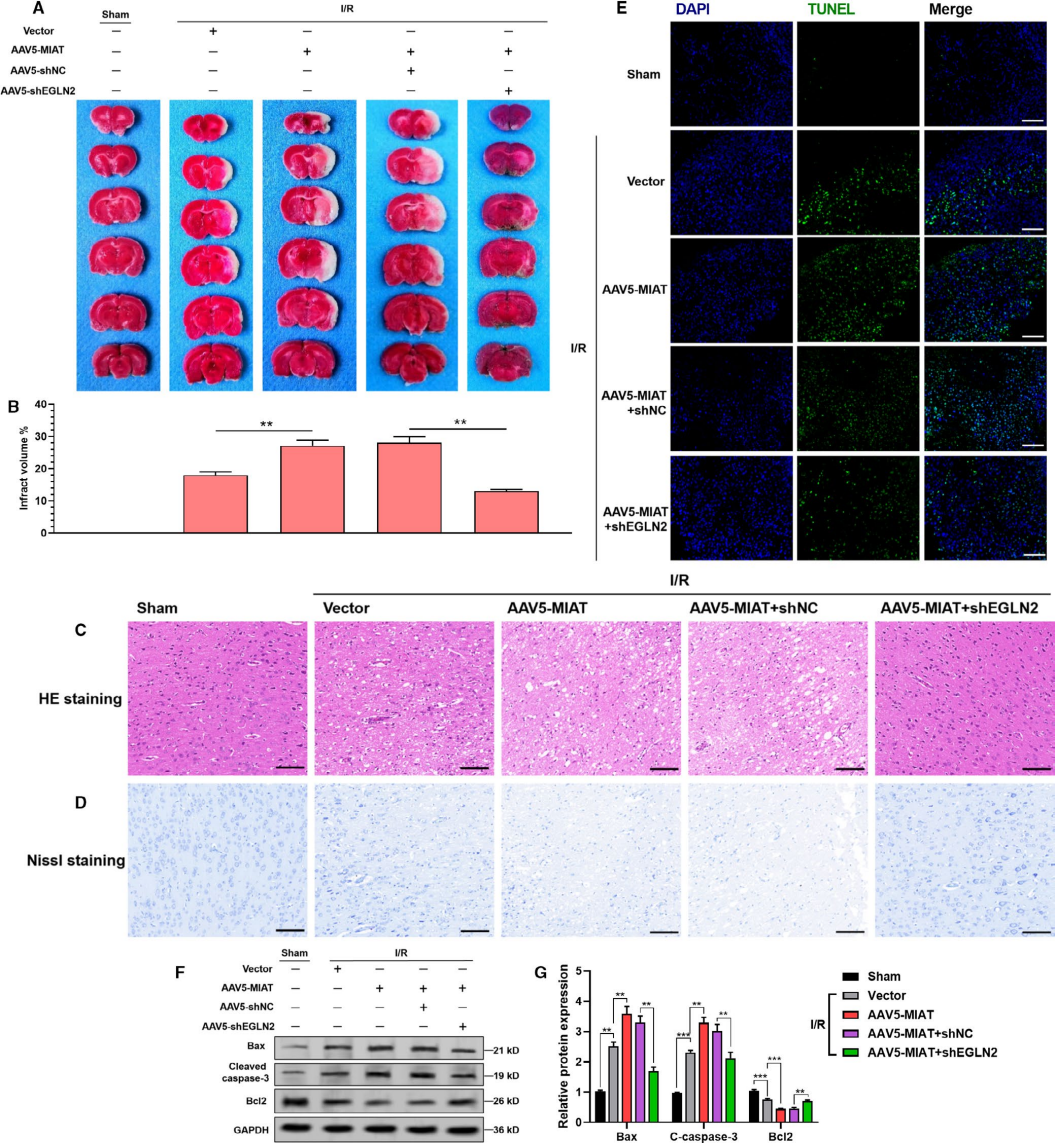
*MIAT*‐EGLN2 axis modulates I/R induced ischaemic brain damage. (A)–(B). Representative photographs (A) and quantitation of infarct area (B) of coronal brain sections stained with TTC, 24 h after I/R injury (*n* = 6). The white‐coloured areas represent the infarct regions in these sections. The rats received stereotactical injection of vector alone (5 × 10^9^ GC in 10 μl), AAV5‐MIAT (5 × 10^9^ GC/10 μl), AAV5‐MIAT (5 × 10^9^ GC/5 μl) +AAV5‐shNC (5 × 10^9^ GC/5 μl) or AAV5‐MIAT (5 × 10^9^/5 μl) +AAV‐shEGLN2 (5 × 10^9^ GC/5 μl). (C)–(E) HE (C), Nissl (D) and TUNEL (E) staining of neuron damage and apoptosis in each group 24 h after I/R injury. Scale bar indicates 100 μm. (F)–(G) Representative images (F) and quantitation (G) of Western blot analysis of Bax, cleaved caspase‐3 (c‐caspase‐3) and Bcl‐2 expression in each group 24 h after I/R injury (*n* = 3)

In the rat cerebral ischaemic model, *MIAT* overexpression enhanced I/R injury, in terms of infarct volume (Figure 5A, 5B), neuron damage (Figure 5C, 5D) and apoptosis (Figure 5E). However, *EGLN2* knockdown abrogated and reversed the detrimental effect of *MIAT* overexpression (Figure 5A–5E). Western blot data confirmed that *MIAT* overexpression enhanced I/R injury‐induced Bax and cleaved caspase‐3 expression but suppressed the expression of Bcl‐2 (Figure 5F, 5G). In comparison, *EGLN2* knockdown significantly alleviated these alterations (Figure 5F, 5G).

### 3.6 *MIAT*‐EGLN2 axis modulates oxidized (GSSG) and reduced (GSH) glutathione ratio in rat neurons

**FIGURE 6:**
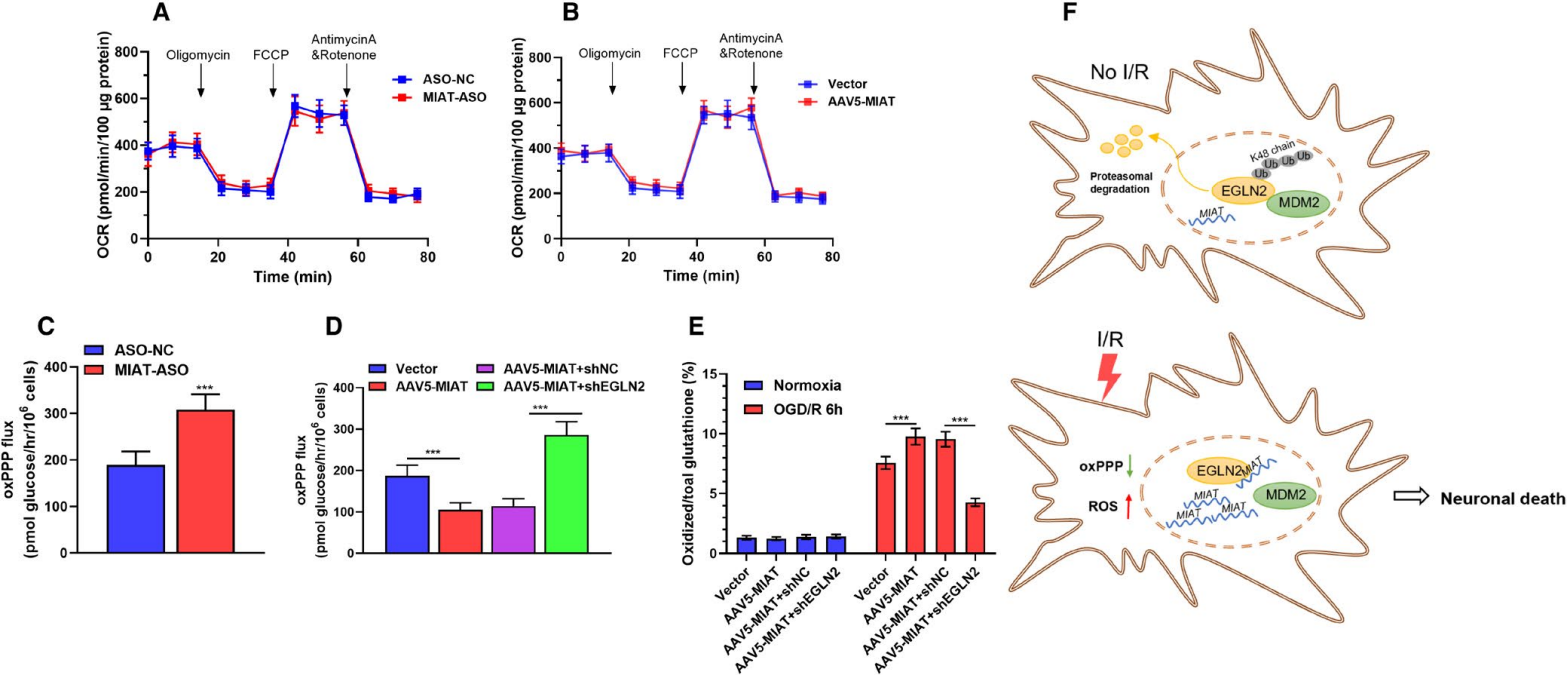
*MIAT*‐EGLN2 axis modulates oxidized (GSSG) and reduced (GSH) glutathione ratio in rat neurons. (A)–(B) OCR was measured in an XF96 Seahorse analyser in primary rat cortical neurons 48 h after *MIAT* knockdown (A) or overexpression (B) (*n* = 3). (C) oxPPP flux in primary rat cortical neurons 48 h after *MIAT* knockdown (*n* = 3). (D) oxPPP flux in primary rat cortical neurons 48 h after *MIAT* overexpression alone or in combination with *EGLN2* knockdown (*n* = 3). (E) Quantitation of GSSG levels as percentage of total glutathione (GSSG +GSH) levels in primary rat cortical neurons 6 h after OGD/R treatment (*n* = 3). Cell treatments were the same as in panel (D). All quantitative data are mean ±SD. (F) Schematic images showing I/R injury‐induced *MIAT* upregulation enhances EGLN2 stability via reducing MDM2 mediated ubiquitin‐proteasomal degradation

One recent study showed that in EGLN2^−/−^ neurons, glycolysis, glucose consumption and glucose oxidation were significantly downregulated. In comparison, the activity of the oxPPP was enhanced.^10^ In the oxPPP pathway, NADPH is generated as a reducing equivalent by glutathione reductase to regenerate GSH, which serves as a critical antioxidant in neurons.^20^ Thus, we further investigated whether the *MIAT*‐EGLN2 axis modulates carbon metabolism in rat neurons. Seahorse XF96 analyzer was utilized to measure the changes in mitochondrial respiration. In primary rat neurons, *MIAT* knockdown or overexpression did not alter mitochondrial respiration (Figure 6A, 6B). In comparison, *MIAT* knockdown increased oxPPP flux, while *MIAT* overexpression decreased the flux (Figure 6C, 6D). *EGLN2* knockdown reversed *MIAT* overexpression induced alteration (Figure 6D). Quantitation of oxidized (GSSG) and reduced (GSH) glutathione 6 h after OGD/R indicated that *MIAT* overexpression increased the ratio of oxidized glutathione, *EGLN2* knockdown drastically reduced the ratio (Figure 6E). Based on these findings, we infer that I/R injury induces *MIAT* upregulation in rat neurons (Figure 6F). *MIAT* enhances EGLN2 stability via reducing MDM2 mediated ubiquitin‐proteasomal degradation (Figure 6F). EGLN2 can reduce oxPPP flux and enhance reactive oxygen species (ROS) generation, leading to neuronal death (Figure 6F).

## 4 DISCUSSION

As a lncRNA involved in I/R injury in both cardiac and cerebral tissues, *MIAT* might participate in complex regulatory networks. It acts as a sponge for miR‐1a in cardiomyocytes and exaggerates acute myocardial infarction via elevating the expression of early growth response gene‐2 (EGR2).^21^ Besides, it promotes p65 nuclear translocation and activates NF‐kappaB signalling, resulting in elevated expression of cleaved‐caspase‐3 and Bax and downregulation of Bcl‐2 in cardiomyocytes after I/R injury.^22^ In cerebral microvascular endothelial cells, it sponges miR‐204 and increases the expression of high mobility group box‐1 protein (HMGB1), leading to enhanced cell apoptosis.^23^ One recent study noticed that in rat cortical neurons, *MIAT* enhances cell autophagy and apoptosis by interacting with Regulated in development and DNA damage responses 1 (REDD1) and reducing its ubiquitination mediated degradation.^8^ This finding suggests that *MIAT* might serve as a protein stabilizer in cortical neurons.

I/R generally increases intraneuronal levels of ubiquitinated proteins, since ischaemia‐derived free radicals can suppress the activities of deubiquitinases.^24^, ^25^ Deubiquitinase inhibition has been considered an endogenous protective mechanism to reduce neuronal proteins from oxidative damage.^24^ However, this opinion is controversial since increased expression and activity of several ubiquitin ligases and associated proteins present neuroprotective effects after cerebral ischaemia, such as STUB1, NEDD4, ITCH and NDFIP1.^25^ STUB1 overexpression exerts neuroprotective functions in neurological diseases via modulating the degradation of some chaperone‐bound proteins.^26^ NDFIP1 binds to PTEN and enhances its ubiquitination mediated by NEDD4 and nuclear import, thereby promoting neuronal survival following cerebral ischaemia.^27^ Therefore, it is necessary to consider the specific functions of ubiquitin ligases in stress regulation and neuronal degeneration.

The current study found that in rat cortical neurons, *MIAT* interacted with EGLN2. Enforced *MIAT* overexpression or knockdown did not alter *EGLN2* mRNA expression. In comparison, *MIAT* increased its stability after I/R injury via reducing its ubiquitin‐mediated degradation. MDM2 is an E3 ligase significantly elevated after transient MCAO^28^ and serves as a neuroprotective protein by promoting p53 degradation and preventing p53‐mediated neuronal apoptotic death.^29^ MDM2 knockdown triggers p53 accumulation and increases neuronal susceptibility to OGD/R‐induced apoptosis.^30^ Similar protect effects of MDM2 were confirmed in primary cultured spinal cord neurons against OGD/R injury.^31^ In this study, we identified EGLN2 as a novel substrate of MDM2. MDM2 interacted with the N‐terminal of EGLN2. It mediated the K48‐linked poly‐ubiquitination of EGLN2, thereby facilitating its proteasomal degradation. The presence of *MIAT* reduced the interaction between MDM2 and EGLN2 and increased EGLN2 stability. By performing *in vivo* MCAO model in rats, we showed that *MIAT* overexpression enhanced infarct volume and induced a higher ratio of neuronal apoptosis. *EGLN2* knockdown significantly reversed the injury. These findings suggest that EGLN2 is an important downstream effector of *MIAT* in I/R injury.

One previous study reported that EGLN2^−/−^ mice had significantly smaller infarct area 24 h after 2 h MCAO, compared to the wild‐type counterparts,^10^ suggesting that EGLN2 exerts critical regulatory effects in I/R injury. However, although EGLN2 is one of the master regulators of the response to hypoxia,^32^ it is the hydroxylation activity of EGLN2 mediates its neuroprotective effect. Knocking down of HIF‐1a, HIF‐2a or HIF‐1b could not abolish the protective effect of PHD1 deficiency against oxygen‐nutrient deprivation.^10^

During the phase of reperfusion, oxidative stress exacerbates ROS production.^33^ The free radicals are highly reactive to a series of molecular targets, including nucleic acids, proteins and unsaturated lipids in cell membranes, generating oxidation‐derived products.^33^ Some endogenous mechanisms are utilized to balance ROS production, including glutathione, coenzyme Q and some other enzymes (superoxide dismutase, glutathione reductase, glutathione‐S‐transferase and glutathione peroxidase). However, when ROS production overwhelms the handing capacity of the antioxidants, cellular damage occurs.^33^ EGLN2 acts as an important regulator of neuronal energy metabolism.^10^ Glucose oxidation is the major energy source of neurons. *EGLN2* knockout does not alter oxygen consumption in neurons but increases oxPPP activity at the expense of glycolysis.^10^ This metabolic alteration enables neurons better against ischaemia via a greater capacity to generate NADPH to scavenge oxygen radicals.^10^ In this study, we found that *MIAT* reduced oxPPP flux and increased oxidized/reduced glutathione ratio via stabilizing EGLN2.

Bioinformatic analysis in this study also observed that SYVN1 and STUB1 are potential E3 ligase interacting with EGLN2. SYVN1 mediates the ubiquitination and degradation of glutathione peroxidase 5 (GPX5), increasing the generation of ROS and apoptosis of cardiomyocytes.^34^ Besides, SYVN1 can enhance I/R induced renal epithelial injury by mediating NRF2 ubiquitylation and degradation.^35^ Furthermore, SYVN1 participates in ER‐associated degradation of unfolded/misfolded proteins, which is linked to brain ischaemia.^36^ Therefore, it is meaningful to explore their potential regulative effect on EGLN2 stability in the future.

In summary, this study identified *MIAT* as a novel stabilizer of EGLN2, via reducing MDM2 mediated K48 poly‐ubiquitination. *MIAT*‐EGLN2 axis exacerbates I/R injury via altering redox homeostasis in neurons. Future studies are required to explore the therapeutic potential of targeting the *MIAT*‐EGLN2 axis to inhibit neuronal apoptotic cell death after acute ischaemic stroke.

## CONFLICT OF INTEREST

The authors have on conflict of interest.

## AUTHOR CONTRIBUTIONS

F. Xu conceived this research; S.‐P. Li, J. Fu, C.‐M Hu and Y. Wang collected, analysed and disposed data; S.‐P. Li, J. Fu, Y. Wang and F. Xu analysed the tables and assembled the figure; S.‐P. Li and F. Xu contributed to all aspects of this study and revised the manuscript for publication.

## Supporting information

Table S1

Figure S1

## Data Availability

The data that support the findings of this study are available from the corresponding author upon reasonable request.
