# Supplementary material for: LncRNA MIAT enhances cerebral ischaemia/reperfusion injury in rat model via interacting with EGLN2 and reduces its ubiquitin‐mediated degradation
Source: J Cell Mol Med. 2021 Oct 22;25(21):10140–51. doi: 10.1111/jcmm.16950 (PMC8572800; doi:10.1111/jcmm.16950)
Supplement: Supplementary file 2 — Figure S1 [file JCMM-25-10140-s002.docx]

**Figure S1. Predicted MDM2 recognizing motif on EGLN2**

| [**Brief information**](http://ubibrowser.ncpsb.org.cn/ubibrowser/home/document/index#SupportingEvidenceBrief) | |
| --- | --- |
| **E3** | [MDM2](http://www.genecards.org/cgi-bin/carddisp.pl?gene=MDM2&keywords=MDM2); [Q00987](http://www.uniprot.org/uniprot/Q00987) |
| **Substrate** | [EGLN2](http://www.genecards.org/cgi-bin/carddisp.pl?gene=EGLN2&keywords=EGLN2); [Q96KS0](http://www.uniprot.org/uniprot/Q96KS0) |
| [**Confidence score**](http://ubibrowser.ncpsb.org.cn/ubibrowser/home/document/index#NomenclatureScore) | 0.765 |
| **Likelihood ratio** | 15.10 |
| [**2. E3 recognizing motif**](http://ubibrowser.ncpsb.org.cn/ubibrowser/home/document/index#SupportingEvidenceDER) | |
| [**Inferred E3 recognition motif**](http://ubibrowser.ncpsb.org.cn/ubibrowser/home/document/index#NomenclatureMotif) | P..QP |
| [**Potential E3 recognition motif**](http://ubibrowser.ncpsb.org.cn/ubibrowser/home/document/index#NomenclatureMotif) | P..QP, P.Q.Q, QPQ, P.SQ, QP.T |
| **Likelihood ratio** | [2.80](http://ubibrowser.ncpsb.org.cn/ubibrowser/home/document/index#NomenclatureLR) |
